# Construction of endothelial cell signatures for predicting the diagnosis, prognosis and immunotherapy response of bladder cancer via machine learning

**DOI:** 10.1111/jcmm.18155

**Published:** 2024-03-01

**Authors:** Yang Fu, Shanshan Sun, Du Shi, Jianbin Bi

**Affiliations:** ^1^ Department of Urology The First Hospital of China Medical University Shenyang Liaoning China; ^2^ Department of Pharmacy The People's Hospital of Liaoning Province Shenyang Liaoning China

**Keywords:** bladder cancer, diagnosis, drug sensitivity, endothelial cell, immune escape, prognosis

## Abstract

We subtyped bladder cancer (BC) patients based on the expression patterns of endothelial cell (EC) ‐related genes and constructed a diagnostic signature and an endothelial cell prognostic index (ECPI), which are useful for diagnosing BC patients, predicting the prognosis of BC and evaluating drug sensitivity. Differentially expressed genes in ECs were obtained from the Tumour Immune Single‐Cell Hub database. Subsequently, a diagnostic signature, a tumour subtyping system and an ECPI were constructed using data from The Cancer Genome Atlas and Gene Expression Omnibus. Associations between the ECPI and the tumour microenvironment, drug sensitivity and biofunctions were assessed. The hub genes in the ECPI were identified as drug candidates by molecular docking. Subtype identification indicated that high EC levels were associated with a worse prognosis and immunosuppressive effect. The diagnostic signature and ECPI were used to effectively diagnose BC and accurately assess the prognosis of BC and drug sensitivity among patients. Three hub genes in the ECPI were extracted, and the three genes had the closest affinity for doxorubicin and curcumin. There was a close relationship between EC and BC. EC‐related genes can help clinicians diagnose BC, predict the prognosis of BC and select effective drugs.

## 1 INTRODUCTION

Among all cancer types, bladder cancer (BC) ranks 13th in mortality rate. A high recurrence rate is an important feature of BC, and long‐term invasive monitoring is needed to detect tumour progression over time.^1^, ^2^ In the past few decades, limited progress has been made in treating BC, and the current main treatment methods for BC are surgery, chemotherapy and immunotherapy.^3^, ^4^ To help clinicians better predict the diagnosis, prognosis and treatment response of BC, tumour molecular markers that are associated with BC should be identified.

The tumour microenvironment (TME) consists of fluids, stromal cells, and immune cells and can interact with tumour cells to promote tumour growth, metastasis, and immune escape.^5^ In our previous study, we found that the stromal cell infiltration level in BC was significantly associated with poor prognosis, suggesting that stromal cells may be an important target for BC therapy.^6^, ^7^ The stromal cells include fibroblasts, endothelial cells (ECs), smooth muscle cells and others. Angiogenesis is regulated by stromal components (mainly ECs) in the microenvironment and can induce cancer metastasis.^8^, ^9^, ^10^, ^11^ The adhesion of tumour cells to ECs can induce the generation of secondary tumours, and tumour ECs interact with tumour cells by secreting proteoglycans to accelerate tumour metastasis.^12^, ^13^ Aberrant angiogenesis promotes hypoxia and mediates immunosuppression and immune escape.^14^, ^15^ Therefore, targeting tumour angiogenesis can be used as a new method for tumour therapy, and ECs, which play an important role, deserve further study.

**FIGURE 1:**
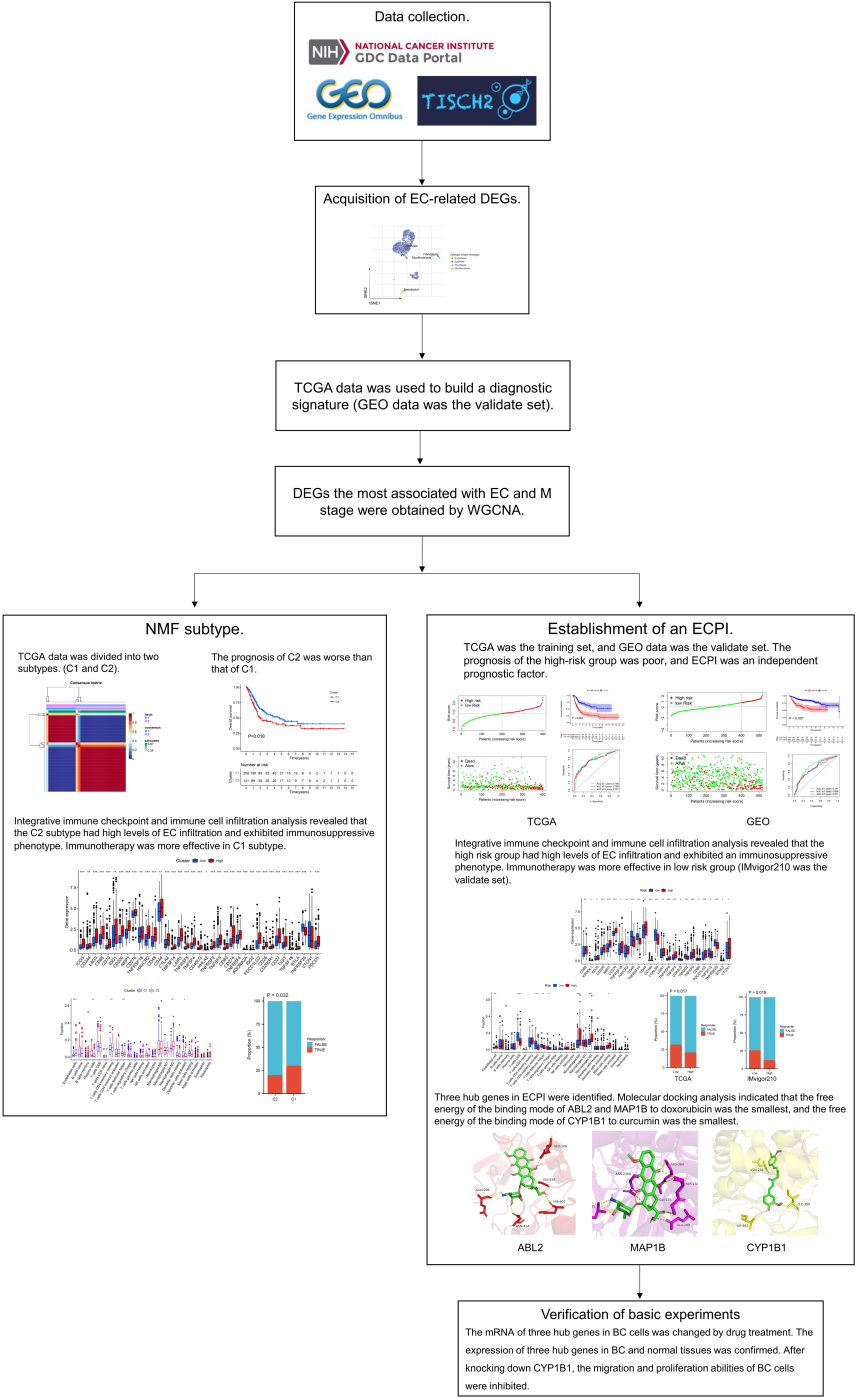
The flow chart. ABL2, ABL proto‐oncogene 2; CYP1B1, cytochrome P450 family 1 subfamily B member 1; DEG, differentially expressed gene; EC, endothelial cell; ECPI, endothelial cell prognostic index; GEO, Gene Expression Omnibus; GSVA, gene set variation analysis; MAP1B, microtubule‐associated protein 1B; NMF, nonnegative matrix factorization; TCGA, The Cancer Genome Atlas; TISCH, TumourTumor Immune Single‐cell Hub; WGCNA, weighted gene coexpression network analysis.

In this study, the roles of ECs and EC‐related genes in BC were comprehensively analysed, and we found that ECs were associated with poor prognosis in BC patients. The level of EC infiltration could significantly affect the immune escape ability of patients with BC. EC‐related genes could be used to classify BC patients and construct diagnostic and prognostic signatures. A summary of the research process and relevant results is shown in Figure 1.

## 2 MATERIALS AND METHODS

### 2.1 Collection and organization of data from multiple public databases

Transcriptional and clinical data from the Gene Expression Omnibus (GEO) (GSE13507, GSE32548 and GSE32894; https://www.ncbi.nlm.nih.gov/geo/) and The Cancer Genome Atlas (TCGA) (TCGA‐BLCA; https://portal.gdc.cancer.gov/) were collected. The three GEO datasets were merged into one dataset, and the Combat algorithm was used to remove batch effects. The mutation data were also downloaded from TCGA‐BLCA for tumour mutational burden (TMB) calculation. The TMB was defined as the total number of substitutions and insertions or deletions per megabase in the exon coding region of a gene in the tumour cell genome. The relevant clinical information from the TCGA‐BLCA and GEO datasets is shown in Tables 1 and 2. Single‐cell expression data from the Tumour Immune Single‐cell Hub (TISCH) database (GSE130001, http://tisch.comp‐genomics.org/) were collected, and the differentially expressed genes (DEGs) in ECs were downloaded.^16^ We defined genes that were significantly differentially expressed in ECs relative to other cells as EC‐related genes. The expression data and clinical data (including immunotherapy efficacy and TMB) of patients in the advanced metastatic BC dataset (IMvigor210) were also collated.

**TABLE 1:** Characteristics of the BC patients obtained from the TCGA database.

Basic information	TCGA (*n* = 409)
Age	68 (median)
Gender	
Female	106
Male	303
Grade	
High	385
Low	21
Unknown	3
Stage	
I & II	132
III & IV	275
Unknown	2
T classification	124
T3 & T4	253
TX	1
Unknown	31
N classification	
N0	237
N1 & N2 & N3	131
NX	36
Unknown	5
M classification	
M0	194
M1	11
MX	202
Unknown	2

Abbreviations: BC, bladder cancer; TCGA, The Cancer Genome Atlas.

**TABLE 2:** Characteristics of the BC patients obtained from the GEO database.

Basic information	GEO (*n* = 598)
Age	70 (median)
Gender	
Female	141
Male	457
Grade	
High	430
Low	168
T classification	
<T2	407
≤T2	191
N classification	
N0	153
N1 & N2 & N3	11
Unknown	434
M classification	
M0	158
M1	7
Unknown	433

Abbreviations: BC, bladder cancer; GEO, Gene Expression Omnibus.

### 2.2 Calculation of EC infiltration

The EC infiltration level in the TCGA‐BLCA dataset was calculated by the extended polydimensional immune characterization (EPIC) algorithm,^17^ microenvironment cell population counter (MCP‐counter) algorithm^18^ and xCell algorithm.^19^ The relationship between the EC infiltration level and disease prognosis was subsequently analysed.

### 2.3 Construction of the diagnostic signature

We used EC‐related genes to construct a diagnostic signature to distinguish BC tissue from normal bladder tissue. The TCGA‐BLCA dataset was defined as the training cohort. The GEO dataset was defined as the validation set. The random forest method was used to rank the importance of EC‐related genes and screen for hub genes (randomForest R package). We chose hub genes with importance scores >4 for further research. The hub genes were used to construct a diagnostic signature. Subsequently, three other machine learning algorithms, namely, the generalized linear model (GLM), eXtreme Gradient Boosting (XGB) and k nearest neighbour (KNN), were applied to validate the diagnostic performance (all algorithms were implemented through the caret R package). To eliminate the impact of the relative imbalance between tumour samples and normal samples in the training set (TCGA‐BLCA) and validation set (GEO), we applied the ROSE algorithm (also via the caret package) when processing the data,^20^ and the area under the curve (AUC) of the receiver operating characteristic curve (ROC, pROC R package) and the area under the precision‐recall curve (AUPRC, PRROC R package) were both used to evaluate the performance of the diagnostic signatures.^21^

### 2.4 Weighted gene coexpression network analysis (WGCNA) for network construction and recognition of hub modules

The gene list downloaded from the TISCH database was used in addition to the TCGA‐BLCA gene expression data and clinical features (including overall survival time, survival status, TNM classification and EC infiltration level calculated according to EPIC) for WGCNA (WGCNA R package) to construct a weighted coexpression network. We converted the adjacency matrix to a topological overlapping matrix to identify the modules via hierarchical clustering, and each module contained at least 30 genes. The correlation coefficients between the modules and clinical data were subsequently calculated to identify the hub clinical modules.

### 2.5 Nonnegative matrix factorization (NMF) for tumour subtypes

We subtyped tumours among the BC patients in the TCGA‐BLCA dataset via the NMF method (NMF R package) using the expression patterns of EC‐related genes according to the WGCNA results by selecting the ‘brunet’ method and performing 50 iterations. The number of clusters k was set to 2 ~ 10, and the minimum member number of each subclass was set to 10. The cophenetic, dispersion and silhouette indicators were used to determine the optimal clustering number, and the optimal clustering number was 2. Additionally, we used other common clustering methods, including hierarchical clustering (sparcl R package) and consensus clustering (ConsensusClusterPlus R package), to compare the effects of clustering. Differences in survival, EC and immune cell infiltration levels, immune checkpoint expression and clinicopathological characteristics between subtypes were further compared. The expression data were uploaded to the Tumour Immune Dysfunction and Exclusion (TIDE, http://tide.dfci.harvard.edu/) website and the TIDE score was calculated online to indirectly evaluate the differences in immunotherapy efficacy between NMF subtypes.

### 2.6 Construction of an EC prognostic index (ECPI) for predicting BC prognosis

Based on the WGCNA results, the gene module most strongly associated with the EC infiltration level was identified. The genes associated with prognosis in the module were screened by univariate Cox analysis based on the TCGA‐BLCA cohort (*p* < 0.01), and the coefficients of each gene included in the ECPI were obtained by least absolute shrinkage and selection operator (LASSO) regression, ridge regression and elastic net (all three algorithms were implemented through the glmnet R package). High‐ and low‐risk groups were distinguished. The predictive power of the ECPI was assessed by AUC of the ROC curve (timeROC R package). Differences in survival rates between the high‐ and low‐risk groups were also compared. The efficacy of the ECPI in independently predicting the prognosis of BC was estimated by univariate and multivariate Cox analyses (survival R package). The nomogram analyses were also performed. (rms R package, clinical features that were statistically significant in the multivariate Cox analysis were included). We also further analysed the relationships of the ECPI with tumour immune parameters and clinicopathological features. The immunotherapy effect of the ECPI was estimated via the TIDE score and verified via the IMvigor210 cohort. The genes included in the ECPI were analysed with a correlation analysis (psych R package), and the genes with the highest number of associations with other genes were defined as the hub genes. Immunohistochemical images of the protein expression of the hub genes were obtained from the Human Protein Atlas (HPA) database (https://www.proteinatlas.org/).

### 2.7 Biological functions and pathways

The biological functions and pathways associated with NMF subtypes and the ECPI were explored via gene set variation analysis (GSVA). The ‘c2.cp.kegg.v7.4.symbols.gmt’ set, ‘c5.all.v7.4.symbols.gmt’ set and ‘c2.cp.reactome.symbols.gmt’ were used to complete the GSVA algorithms.

### 2.8 Drug sensitivity and molecular docking

The DEGs between NMF subtypes were uploaded to the Connectivity Map (CMap, https://clue.io/data) website for online analysis to explore potential small‐molecule drugs. The connection between the ECPI and the half‐maximal inhibitory concentration (IC50) was analysed with a |correlation coefficient| >0.35 as the cutoff value (pRRophetic R package). The specific protein structures of the hub genes screened by correlation analysis were obtained from the Protein Data Bank (PDB) database (https://www.rcsb.org/), and the protein structures not included in the PDB database were constructed using AlphaFold 2. The chemical substances significantly related to the hub genes in BC were downloaded from the Comparative Toxicogenomics Database (CTD) (http://ctdbase.org/).^22^ In the PubChem database (https://pubchem.ncbi.nlm.nih.gov/), the 3D structures of the chemical substances were collected. AutoDock Vina software 1.5.7 was used to perform molecular docking to evaluate the binding mode of the hub genes and chemical substances. Binding modes capable of producing stable hydrogen bonds were identified as effective binding. The molecular docking data were visualized with LigPlus software (2D) and PyMOL software (education version) (3D).^23^

### 2.9 Patient tissue sample collection

This study was approved by the Ethics Committee of the First Hospital of China Medical University (Approval No. [2021]121). Each patient signed a consent form allowing the use of the sample in our study. We collected BC tissue and paired normal tissue samples from 30 patients who underwent total cystectomy between January 2023 and August 2023. All the samples were maintained at −80°C before analysis.

### 2.10 Cell culture and transfection

The human BC cell lines T24 and UMUC3 were obtained from the National Certified Cell Culture Collection Center (China). The cells were grown in RPMI 1640 medium (Procell, China) and DMEM (Procell, China) supplemented with 10% fetal bovine serum (FBS; Procell, China) in a humidified incubator at 37°C and 5% CO_2_. Small interfering RNA (siRNA) targeting CYP1B1 was synthesized by JTS Scientific (China). The siRNAs were transfected into cells using Lipofectamine 3000 (Invitrogen, USA) according to the manufacturer's instructions. Total RNA was extracted 24 h after transfection. Short hairpin RNA (shRNA; Genechem, China) was used to construct UMUC3 cells with CYP1B1 knockdown. Puromycin (5 μg/mL) was used for selection, and quantitative real‐time (qRT)‐PCR was used to confirm the knockdown efficiency.

### 2.11 RNA extraction and qRT–PCR

Total RNA was extracted from all tissues and BC cells. Total RNA was extracted using TRIzol (Invitrogen, USA), and cDNA was synthesized using the PrimeScriptTM RT Reagent Kit (Takara, Japan). Subsequent qRT–PCR was performed using SYBR Premix Ex Taq reagent (Takara, Japan) via the LightCycler 480 II system. The data were calculated using the 2^−ΔΔCT^ method and normalized to the expression of GAPDH. The primer sequences are shown in Table 3.

**TABLE 3:** The list of primers.

Gene	Primer
CYP1B1	Forward, 5’‐GCTGCAGTGGCTGCTCCT‐3′ Reverse, 5’‐CCCACGACCTGATCCAATTCT‐3′
ABL2	Forward, 5’‐GTTGAACCCCAGGCACTAAAT‐3′ Reverse, 5’‐CAATGAAGAGATTAGGGTCACTC‐3′
MAP1B	Forward, 5’‐TCCGACACTTAGACCGAGTGG −3′ Reverse, 5’‐ACATGCTGTTTATTCCAGGCAA‐3’
GAPDH	Forward, 5’‐GGCTGTTGTCATACTTCTCATGG‐3′ Reverse, 5′‐ GGAGCGAGATCCCTCCAAAAT‐3’
CYP1B1 si1	Forward, 5’‐CCACUAUCACUGACAUCUUTT‐3′ Reverse, 5’‐AAGAUGUCAGUGAUAGUGGTT‐3’
CYP1B1 si2	Forward, 5’‐GCGAAGAACUUUCUAAGAUTT −3′ Reverse, 5’‐AUCUUAGAAAGUUCUUCGCTT −3’

Abbreviations: ABL2, ABL proto‐oncogene 2; CYP1B1, cytochrome P450 family 1 subfamily B member 1; GAPDH, glyceraldehyde‐3‐phosphate dehydrogenase; MAP1B, microtubule associated protein 1B.

### 2.12 CCK‐8 detection

Cell viability was assessed using CCK‐8 reagent (Sellect, USA). T24 and UMUC3 cells transfected with CYP1B1 siRNA or negative control (NC) were plated at 1.0 × 10^3^ cells/well and then cultured in 96‐well plates for 4 days. After adding CCK‐8 reagent at the same time point every day and incubating for 1 h, the optical density was measured at 450 nm.

The CCK8 reagent was also used to test the toxicity of doxorubicin (Sellect, USA) and curcumin (Sellect, USA) on BC cells. UMUC3 and T24 cancer cells were seeded in 96‐well plates at a density of 5000 cells/well. Five different concentrations of doxorubicin and curcumin were added to the wells, which were subsequently cultured for 48 h. After adding CCK‐8 and incubating for 1 h, the optical density was measured.

### 2.13 Colony formation assay

T24 and UMUC3 cells transfected with CYP1B1 siRNA or NC were placed in six‐well plates (1 × 10^3^ cells/well) and maintained for 14 days. Colonies were stained with 0.1% crystal violet (Beyotime, China).

### 2.14 Transwell assay

A total of 600 μL of culture medium containing 10% FBS was added to the 24‐well plate. T24 and UMUC3 cells transfected with CYP1B1 siRNA or NC were suspended in 200 μL of serum‐free medium and plated in the upper chamber of the Transwell chamber (Corning, USA) for 24 h. The cells in the lower chamber were counted after staining with 0.1% crystal violet solution for 15 min (Beyotime, China).

### 2.15 Real‐time cell analysis (RTCA)

Transfected T24 and UMUC3 cells were seeded in E plates at a density of 1 × 10^3^ cells/well for 70 h. The RTCA xCELLigence S16 automatically records cell growth curves. Cell numbers were normalized to common BC cell attachment times (4 h).

### 2.16 Xenograft experiments

Xenograft experiments in nude mice (BALB/c nude mice, female, 5 weeks old) were conducted at the Experimental Animal Center of China Medical University. Twelve mice were randomly divided into two groups. Each mouse in the control group was injected subcutaneously with 3 × 10^6^ UMUC3 NC cells, and each mouse in the knockdown group was injected subcutaneously with 3 × 10^6^ UMUC3 cells stably transfected with CYP1B1 knockdown lentivirus. Tumours were harvested 4 weeks later. The xenograft experiments were approved by the Animal Care and Use Committee.

### 2.17 Statistical analysis

The Wilcoxon rank sum test was performed to assess the differences between groups for the nonnormally distributed data. The *t* test was applied to normally distributed data. The immune cell infiltration level was determined using cell type identification via the estimating relative subsets of RNA transcripts (CIBERSORT) algorithm.^24^ The estimation of stromal and immune cells in malignant tumour tissues was performed using the expression data (ESTIMATE) algorithm to calculate TME‐related parameters.^25^ The chi‐square test was used for statistical analysis of the enumeration data. Correlation analysis was performed via Spearman's test. All the above operations were implemented with R software 4.2.1 and GraphPad Prism 9.0.0.

## 3 RESULTS

### 3.1 A high level of EC infiltration indicated a worse prognosis in BC patients

The effect of EC infiltration levels on BC prognosis was calculated with three different algorithms (EPIC, MCP‐counter and xCell) in the TCGA‐BLCA cohort, and the results revealed that a greater level of EC infiltration was significantly related to a worse prognosis of BC (Figure S1A–C).

### 3.2 Gene screening for differential expression in ECs compared with other cell types

Cell cluster (Figure S1D) and cell annotation (Figure S1E) data from the GSE130001 dataset were downloaded directly from the TISICH database. A total of 1922 genes differentially expressed in ECs relative to other cell types were identified (|log(FC)| >0.5, *p* < 0.05 as criteria).

### 3.3 Construction of the diagnostic signature

**FIGURE 2:**
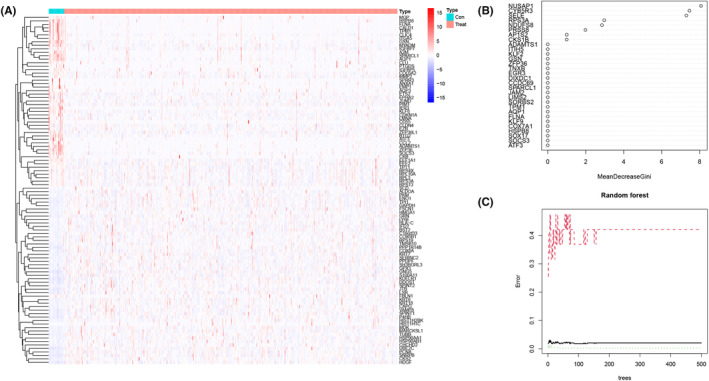
Diagnostic signature. The expression patterns of the top 50 genes among the 885 genes were visualized via a heatmap (A). The importance score of each gene was obtained by the random forest method, and the genes included in the diagnostic signature were NUSAP1, CYB5R3 and SELE (B, C). CYB5R3, cytochrome b5 reductase 3; NUSAP1, nucleolar and spindle associated protein 1; SELE, selectin E.

We used EC‐related DEGs to construct a diagnostic signature to distinguish BC tissue from normal bladder tissue. A total of 885 genes that were also differentially expressed between BC tissues and normal bladder tissues were identified as DEGs in ECs (|log(FC)| >2, *p* < 0.05 as criteria) from the TCGA‐BLCA cohort. The expression patterns of the top 50 genes were visualized with a heatmap (Figure 2A). The importance score of each gene was obtained by the random forest method, and the genes included were nucleolar and spindle associated protein 1 (NUSAP1), cytochrome B5 reductase 3 (CYB5R3) and selectin E (SELE) (Figure 2B,C). The diagnostic signature was constructed with the RF, GLM, XGB and KNN algorithms, and the AUC and AUPRC values are shown in Table 4. When the GEO dataset was used for validation, the diagnostic signature remained robust (also shown in Table 4).

**TABLE 4:** The AUC and AUPRC of multiple machine learning algorithms.

Algorithms	AUC	AUPRC
RF (TCGA)	0.985	0.960
RF (GEO)	0.855	0.900
GLM (TCGA)	0.992	0.991
GLM (GEO)	0.855	0.907
XGB (TCGA)	0.990	0.914
XGB (GEO)	0.842	0.903
KNN (TCGA)	0.984	0.949
KNN (GEO)	0.841	0.904

Abbreviations: AUC, the area under the curve; AUPRC, the area under the precision‐recall curve; GEO, the Gene Expression Omnibus; GLM, generalize linear model; KNN, k nearest neighbour; RF, random forest; TCGA, the Cancer Genome Atlas; XGB, eXtreme Gradient Boosting.

### 3.4 WGCNA

DEGs were subjected to further WGCNA. When the threshold was set to 10, the average number of network connections was close to that of the scale‐free network (Figure S2A,B). The following hierarchical clustering procedure created a gene clustering tree, which divided the 1922 EC‐related genes into eight coexpression modules (Figure S2C). According to the heatmap results of the correlation between clinical traits and modules, we selected the green module that had the most significant relationships with both ECs and the M stage of BC pathogenesis for subsequent research (Figure S2D).

### 3.5 Identification of NMF subtypes

**FIGURE 3:**
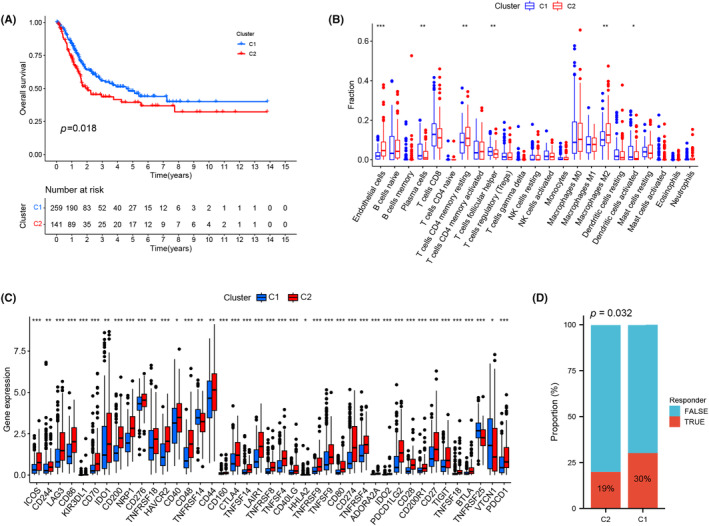
Further analysis of NMF. The disease prognosis in C2 was significantly worse than that of C1 (A). C2 had a greater infiltration level in the EC than C1 did, and activated memory CD4 T cells and M2 macrophages were enriched in C2, while plasma cells, follicular helper T cells and activated dendritic cells were enriched in C1 (B). In C2, the expression level of immune checkpoint genes leading to immune escape was significantly increased (only immune checkpoints with statistical differences were displayed), indicating that C2 had an immunosuppressive microenvironment (C). The immunotherapy effect of C1 was better than that of C2 (D). EC, endothelial cell; NMF, nonnegative matrix factorization. *p < 0.05; **p < 0.01; ***p < 0.001.

A total of 196 genes in the green module that were also differentially expressed between BC tissues and normal bladder tissues (|log(FC)| >2, *p* < 0.05 as criteria) were identified as candidate genes. After comprehensive consideration, *K* = 2 was selected as the number of subgroups, and there was a clear boundary between the NMF subtypes (C1 and C2) in the heatmap (Figure S3A–I). C2 patients had a worse disease prognosis and greater EC infiltration than C1 patients (Figure 3A). M2 macrophages were enriched in C2, while plasma cells, follicular helper T cells and activated dendritic cells were enriched in C1 (Figure 3B). In C2, the expression levels of immune checkpoint molecules were significantly increased (only immune checkpoints with statistical differences were displayed), indicating that C2 had an immunosuppressive microenvironment (Figure 3C). Due to the weaker level of immunosuppression, the immunotherapy effect of C1 was better than that of C2 (Figure 3D). In addition, C2 was significantly associated with advanced clinical stage, T stage and N stage in BC patients (Figure S3J). The top 10 potential small‐molecule drugs screened by CMap and related descriptions are listed in Table 5. Biological functions and pathways enriched in the C1 and C2 subtypes after GSVA were visualized [Gene Ontology (GO) (Figure S3K), Kyoto Encyclopedia of Genes and Genomes (KEGG) (Figure S3L) and Reactome (Figure S5M)]. The hierarchical clustering and consensus clustering methods were also applied, but the results were not satisfactory, indicating that the NMF method had unique advantages (Figure S4A–M).

**TABLE 5:** The top 10 small molecule drug screening based on CMap. (Ranked by database‐calculated score).

Drugs	Score	Description
Merck60	98.38	HDAC inhibitor
XMD‐892	93.09	MAP kinase inhibitor
Periplocymarin	85.17	Apoptosis stimulant
HC‐toxin	83.48	HDAC inhibitor
BI‐2536	83.12	PLK inhibitor
Anisomycin	80.91	DNA synthesis inhibitor
Cephalotaxine	80.65	Protein synthesis inhibitor
Maprotiline	79.73	Norepinephrine reuptake inhibitor
Trichostatin‐a	77.83	HDAC inhibitor
Perhexiline	76.11	Carnitine palmitoyltransferase inhibitor

Abbreviations: CMap, connectivity map; HDAC, histone deacetylase; MAP, mitogen‐activated protein; PLK, polo like kinase.

### 3.6 Establishment of the ECPI

**FIGURE 4:**
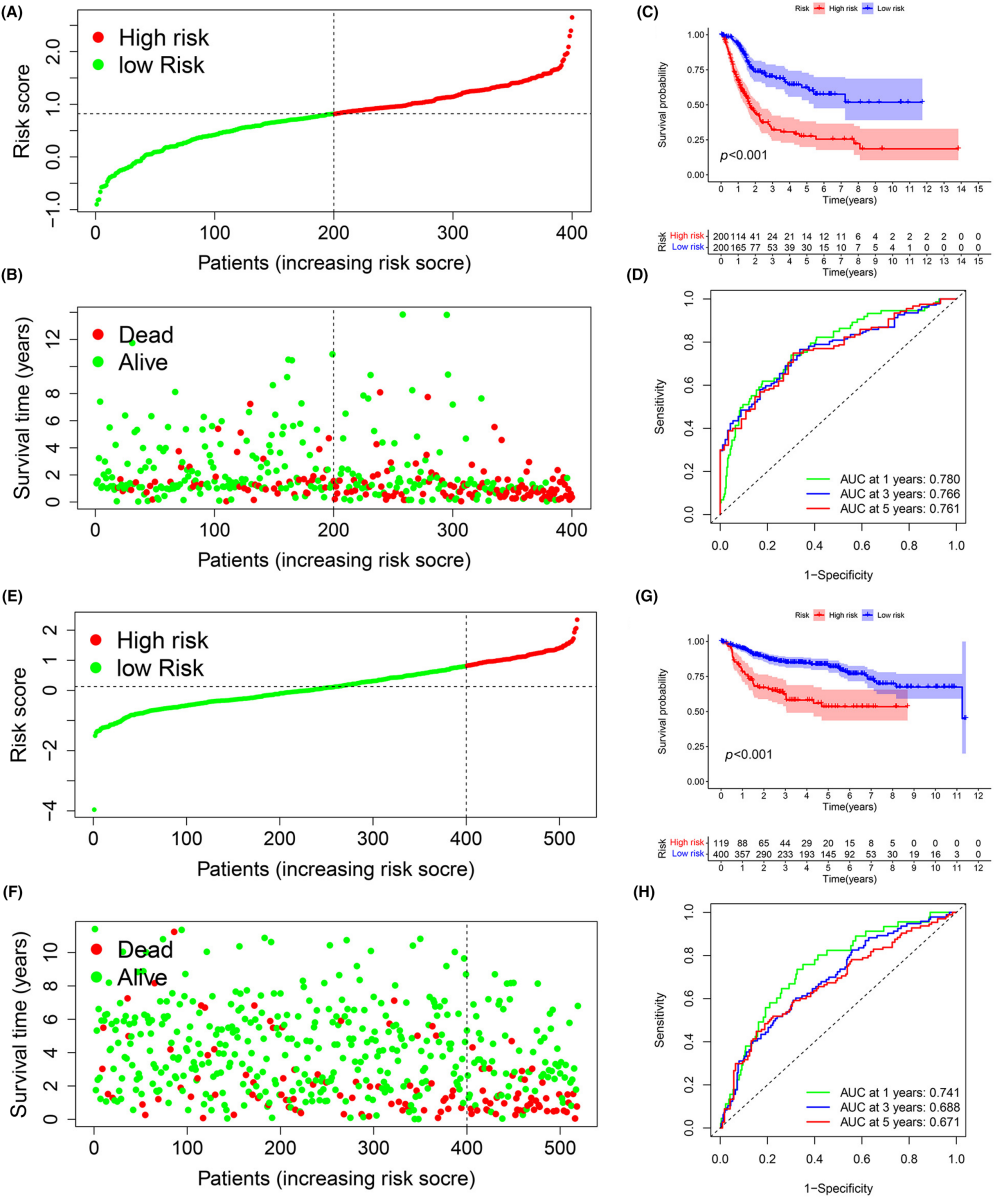
The prognostic risk signature (ECPI). LASSO regression was used to construct a prognostic risk signature, and the coefficients of the included genes were obtained to calculate the risk score. The high‐ and low‐risk groups were divided on the basis of the median risk score (A, B). The high‐risk group had a poorer disease prognosis than the low‐risk group did (C). The AUC of the ROC curve for the risk signature was 0.780 (1 year), 0.766 (3 years) and 0.761 (5 years) (D). The GEO data were used as the validation set, and patients were divided into high‐ and low‐risk groups (E, F). The high‐risk group also had a poorer disease prognosis than the low‐risk group did (G). The AUC of the ROC curve for the risk signature was 0.741 (1 year), 0.688 (3 years) and 0.671 (5 years) (H). AUC, the area under curve; ECPI, endothelial cell prognostic index; GEO, Gene Expression Omnibus; LASSO, least absolute shrinkage and selection operator; ROC, receiver operating characteristic curve.

The genes included in the green module were also used to construct the ECPI. Among the genes in the green module, prognosis‐related genes were screened via univariate Cox analysis of the TCGA‐BLCA cohort (Table S1). Subsequently, LASSO regression, elastic net (Figure S5A–F) and ridge regression (Figure S5G–L) were used to construct the ECPI, and the coefficients of the included genes were obtained to complete the calculation of the ECPI. Later, the results of the three algorithms were similar, but the ECPI constructed with LASSO was more concise. Therefore, we used the LASSO algorithm for further analysis (the signature constructed by the LASSO algorithm incorporated 17 genes, the signature constructed by the elastic net algorithm incorporated 18 genes and the signature constructed by the ridge regression algorithm incorporated 54 genes). The high‐ and low‐risk groups were divided on the basis of the median value of the ECPI (Figure 4A,B). The overall survival rate of the high‐risk group was worse than that of the low‐risk group (Figure 4C). The AUC values of the ROC for the ECPI were 0.780 (1 year), 0.766 (3 years) and 0.761 (5 years) (Figure 4D). A higher ECPI was also related to age, clinical stage, T stage, and N stage (Figure S6A–D), regardless of sex and M stage (Figure S6E,F). The GEO dataset was used for validation (Figure 4E,F). The overall survival rate of the high‐risk group was worse than that of the low‐risk group in this dataset (Figure 4G). The AUC values of the ROC curve for the ECPI were 0.741 (1 year), 0.688 (3 years) and 0.671 (5 years) (Figure 4H). Both univariate Cox analysis and multivariate Cox analysis demonstrated that the ECPI score was an independent factor for predicting the prognosis of BC in the TCGA‐BLCA (Figure S6G,H) cohort and GEO cohort (Figure S6I,J) (because there were only a few samples in the GEO cohort that included N classification and M classification, these two clinical characteristics were not included in the statistical analysis of the GEO cohort). The nomogram established by the ECPI illustrated that the ECPI could be used to assess the prognosis of BC at 1, 3, and 5 years in the TCGA‐BLCA cohort (Figure S7A–D) and GEO cohort (Figure S7E–H).

**FIGURE 5:**
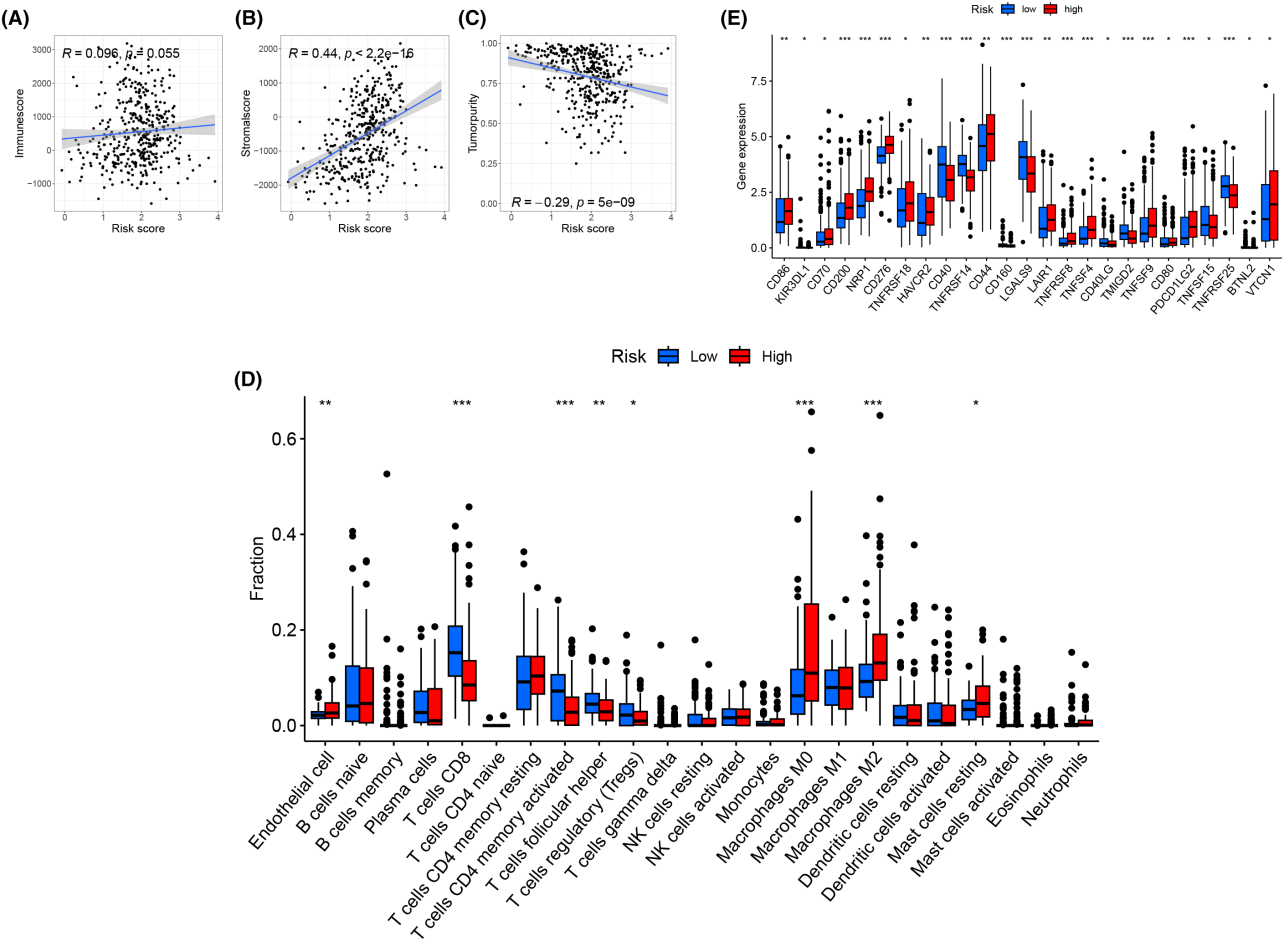
The relationship between tumour immune‐related parameters and the ECPI As the ECPI increased, the immune score did not change significantly, while the stromal score increased, leading to a decrease in tumour purity (A–C). Enrichment of various types of T cells was found in the low‐risk group, whereas enrichment of mainly macrophages was found in the high‐risk group (D). Furthermore, the low‐risk group had lower expression levels of immune checkpoint genes (E, only immune checkpoints with statistical differences were displayed). ECPI, endothelial cell prognostic index; TMB, tumour mutational burden. *p < 0.05; **p < 0.01; ***p < 0.001.

The relationships between tumour immune‐related parameters and the ECPI were further analysed. As the ECPI increased, the immune score did not change significantly, but the stromal score increased, leading to a decrease in tumour purity (Figure 5A–C). Enrichment of various types of T cells was found in the low‐risk group, whereas enrichment of mainly macrophages and ECs was found in the high‐risk group (Figure 5D). Furthermore, the low‐risk group had lower expression levels of immune checkpoint genes (Figure 5E, only immune checkpoints with statistical differences were displayed), higher TMB (Figure S8A) and better immunotherapy outcomes (TIDE score, Figure S8B). The results of TMB and immunotherapy were also validated using the IMvigor210 dataset (Figure S8C,D).

By analysing the connection between the ECPI and drug sensitivity, the top 10 drugs according to correlation coefficients were determined and are shown in Figure S8. The sensitivities of WH‐4‐023 and TGX221 were positively correlated with the ECPI (Figure S8E,F), and the remaining sensitivities were negatively correlated (Figure S8G–N).

Through coexpression analysis, ABL proto‐oncogene 2 (ABL2), cytochrome P450 family 1 subfamily B member 1 (CYP1B1) and microtubule associated protein 1B (MAP1B) were found to be hub genes in the ECPI, which deserved further study (Figure S9A). The protein expression of the three hub genes was analysed by using the HPA database, and these proteins were found to be highly expressed in BC tissues (Figure S9B‐G).

Finally, GSVA was performed to analyse the relevant biological functions of the genes associated with the ECPI. The GO (Figure S9H), KEGG (Figure S9I) and Reactome (Figure S9J) results were visualized.

### 3.7 Molecular docking

**FIGURE 6:**
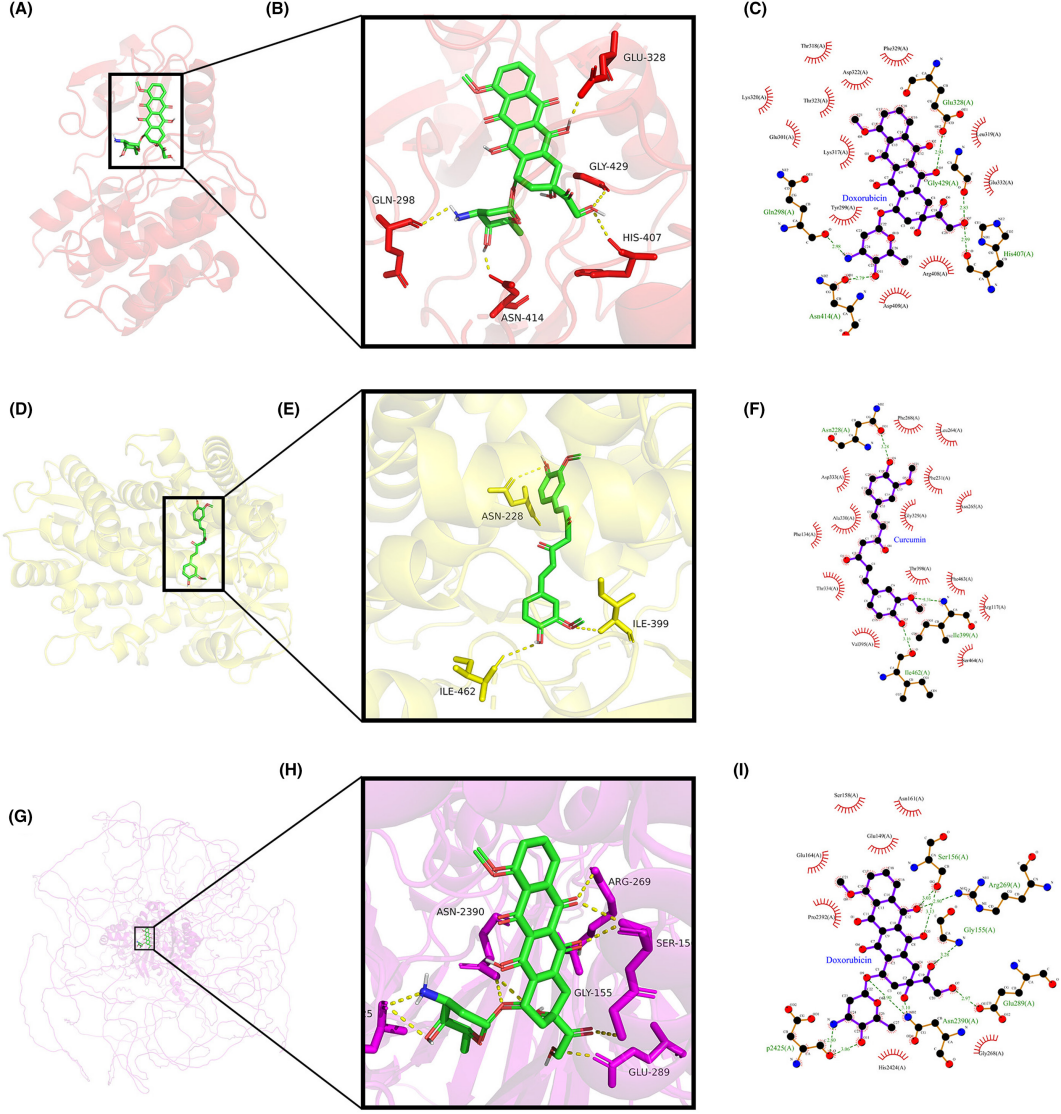
Molecular docking. The binding modes of the minimum free energy docked compounds ABL2 (doxorubicin) (A–C), CYP1B1 (curcumin) (D–F) and MAP1B (doxorubicin) (G–I) were visualized. ABL2, ABL proto‐oncogene 2; CYP1B1, cytochrome P450 family 1 subfamily B member 1; MAP1B, microtubule‐associated protein 1B.

**FIGURE 7:**
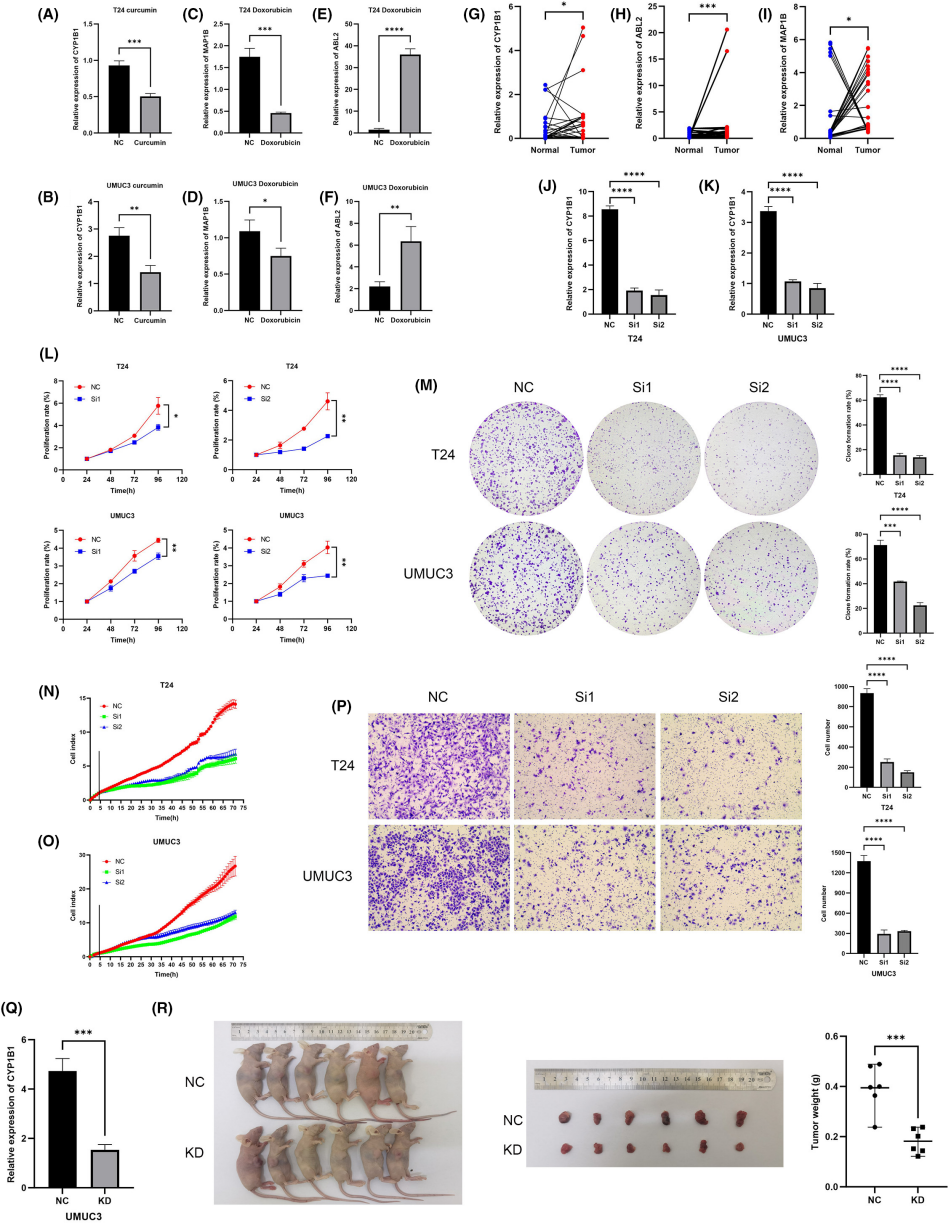
Verification of the basic experiments. After treatment with curcumin at the IC50, the CYP1B1 mRNA level in BC cells was significantly reduced (A, B). After treatment with doxorubicin at the IC50, the MAP1B mRNA level in BC cells was significantly decreased (C, D), and the ABL2 mRNA level was significantly increased (E, F). By analysing BC tissues and paired normal tissues from 30 patients, we confirmed that the mRNA expression of CYP1B1, ABL2 and MAP1B in BC tissues was significantly greater than that in normal tissues (G–I). CYP1B1 mRNA was significantly reduced in BC cells after transfection (J, K). CCK‐8 (L), colony formation (M) and RTCA (N, O) assays confirmed that the proliferation of BC cells with reduced CYP1B1 expression was significantly decreased. Transwell assays showed that the migration ability of BC cells was significantly decreased after transfection (P). After stable transfection of the CYP1B1 knockdown lentivirus, the mRNA level in UMUC3 cells was confirmed to be significantly reduced (Q). Compared with those in the control group, the tumours that formed when CYP1B1 knockdown UMUC3 cells were injected subcutaneously into nude mice were smaller in size and lighter in weight (R). ABL2, ABL proto‐oncogene 2; BC, bladder cancer; CYP1B1, cytochrome P450 family 1 subfamily B member 1; IC50, half‐maximal inhibitory concentration; KD, knockdown; NC, negative control; RTCA, real‐time cell analysis.*p < 0.05; **p < 0.01; ***p < 0.001; ^****^ *p* < 0.0001.

The top five molecular docking results for each gene are listed in Table 6. The binding modes of the minimum free energy docked compounds ABL2 (doxorubicin) (Figure 6A–C), CYP1B1 (curcumin) (Figure 6D–F) and MAP1B (doxorubicin) (Figure 6G–I) were also visualized. According to the results of the CCK‐8 assay, the IC50s of doxorubicin on T24 and UMUC3 cells were 0.678 μM and 0.646 μM, respectively. The IC50s of curcumin on T24 and UMUC3 were 22.780 μM and 13.400 μM, respectively. After treatment with curcumin at the IC50, the CYP1B1 mRNA level in BC cells was significantly reduced (Figure 7A,B). After treatment with doxorubicin at the IC50, the MAP1B mRNA level in BC cells was significantly decreased (Figure 7C,D), and the ABL2 mRNA level was significantly increased (Figure 7E,F). These results confirmed the association of doxorubicin and curcumin with the three hub genes.

**TABLE 6:** The top five molecular docking results for each gene.

Gene	Pubchem ID	Chemical substance	Free energy
ABL2	Conformer3D_CID_31703	Doxorubicin	−8.2
ABL2	Conformer3D_CID_644019	Cannabidiol	−7.8
ABL2	Conformer3D_CID_3715	Indomethacin	−7.2
ABL2	Conformer3D_CID_4763	Phenobarbital	−7.1
ABL2	Conformer3D_CID_1983	Acetaminophen	−6.5
CYP1B1	Conformer3D_CID_969516	Curcumin	−10.4
CYP1B1	Conformer3D_CID_5991	Ethinylestradiol	−9.9
CYP1B1	Conformer3D_CID_3973	LY294002	−9.8
CYP1B1	Conformer3D_CID_4055	Menadione	−9.5
MAP1B	Conformer3D_CID_31703	Doxorubicin	−8.6
MAP1B	Conformer3D_CID_5288209	Fenretinide	−8.5
MAP1B	Conformer3D_CID_5280343	Quercetin	−8.3
MAP1B	Conformer3D_CID_2236	Aristolochic acid	−7.3
MAP1B	Conformer3D_CID_644019	Cannabidiol	−7.2

Abbreviations: ABL2, ABL proto‐oncogene 2; CYP1B1, cytochrome P450 family 1 subfamily B member 1; MAP1B, microtubule associated protein 1B.

### 3.8 Knockdown of CYP1B1 inhibited the proliferation and migration of BC cells

By analysing BC tissues and paired normal tissues from 30 patients, it was confirmed that the mRNA expression of CYP1B1, ABL2 and MAP1B in BC tissues was significantly greater than that in normal tissues (Figure 7G–I). Among the three hub genes, two genes (MAP1B and ABL2) have been reported to have an impact on the malignant traits of BC cells^26^, ^27^; therefore, we selected CYP1B1 as the target for subsequent experimental verification. CYP1B1 mRNA was significantly reduced in BC cells after transfection (Figure 7J,K). CCK‐8 (Figure 7L), colony formation (Figure 7M) and RTCA (Figure 7N,O) assays confirmed that the proliferation of BC cells with reduced CYP1B1 expression was significantly decreased. Transwell assays showed that the migration ability of BC cells was significantly decreased after transfection (Figure 7P). After stable transfection of the CYP1B1 knockdown lentivirus, the CYP1B1 mRNA level in UMUC3 cells was confirmed to be significantly reduced (Figure 7Q). Compared with those in the control group, the tumours that formed when CYP1B1 knockdown UMUC3 cells were injected subcutaneously into nude mice were smaller in size and lighter in weight (Figure 7R).

## 4 DISCUSSION

Based on our previous studies, we determine that BC stromal cells might be closely related to the development of tumours. In tumours, nonmalignant stromal cells also exhibit abnormal phenotypes and secrete tumour‐promoting factors to accelerate tumour progression.^28^ In the microenvironment of malignant tumours, ECs have high proangiogenic activity and activate the expression of angiogenic genes.^29^ The newly formed tumour blood vessels exhibit obvious abnormalities in morphology, and the connections between ECs become loose, which makes it easy for tumour cells to penetrate tumour blood vessels and cause tumour metastasis.^30^ Therefore, targeting EC metabolism to achieve antiangiogenic effects and vascular normalization could be a promising approach for tumour therapy.^31^, ^32^

We constructed a diagnostic signature via the random forest method using EC‐related genes obtained from the TISCH database. The results suggested that the diagnostic signature could accurately distinguish BC tissue from normal bladder tissue. A total of three genes were included in the diagnostic signature (NUSAP1, CYB5R3 and SELE). NUSAP1 is highly expressed in BC tissue and can promote BC progression through the TGF‐β signalling pathway.^33^ In oestrogen receptor‐negative breast cancer, CYB5R3 activates metastasis formation and is significantly associated with poor prognosis.^34^ Moreover, inhibition of SELE on the EC surface can produce an antivascular effect, thereby inhibiting the growth of hepatocellular carcinoma.^35^

Using WGCNA, we then screened out the green module that was most significantly connected with ECs. The genes in the green module were used for the identification of tumour subtypes by NMF and for the construction of the ECPI. Compared to traditional clustering and dimensionality reduction methods, NMF has been shown to identify subtle, context‐dependent biological patterns and was less sensitive to the selection and/or perturbation of the input genes used in factorization. Standard clustering methods cannot capture this context dependence.^36^ The NMF results revealed significant differences in prognosis, EC and immune cell infiltration, immune checkpoint expression, clinical trait information and GSVA among the tumour subtypes. The results of multiple datasets collectively demonstrated that the C2 subtype with higher levels of EC infiltration was an immunosuppressive subtype with a poor response to immunotherapy. A total of 17 genes were included in the ECPI using LASSO. Patients in the high‐risk group had a worse disease prognosis, and the ECPI was considered an independent prognostic factor. The reason why the ECPI did not affect the immune score was that immunosuppressive immune cells (M2 macrophages) aggregated in the high‐risk group, while immune effector cells (CD8 T cells) clustered in the low‐risk group, resulting in little change in the immune score. The high‐risk group was more sensitive to two drugs, WH‐4‐023 [sarcoma (SRC) kinase inhibitor] and TGX221 [phosphoinositide 3 kinase (PI3K) inhibitor], indicating that the pathogenesis of the high‐risk group was associated with SRC kinase and PI3K signalling.^37^, ^38^ Through GSVA, we found that the genes in the high‐risk group were enriched mainly in angiogenesis, stromal cells, M2 macrophages, the extracellular matrix (ECM) and glucose metabolism. Stromal cells and M2 macrophages can achieve immunosuppression and immune escape through various mechanisms.^39^, ^40^, ^41^ Elevated glucose metabolism promotes the proliferation of BC cells and induces BC recurrence and metastasis.^42^, ^43^ Dysregulation of the ECM can disrupt tissue homeostasis, which can facilitate the occurrence and progression of tumours.^44^, ^45^ In addition, signalling pathways common in BC (WNT, TGF‐β and MAPK pathways) were also enriched in the high‐risk group.^46^, ^47^, ^48^

Three hub genes were identified via coexpression analysis of these 17 genes (ABL2, MAP1B and CYP1B1). There are currently reports showing that highly expressed ABL2 and MAP1B promote the malignant behaviour of BC cells, but the effect of CYP1B1 on BC cells is unknown. We verified the high expression of the three hub genes in BC tissues, and knocking down CYP1B1 weakened the proliferation and migration abilities of BC cells. Through molecular docking analysis, we confirmed that two drugs, curcumin and doxorubicin, were related to the three hub genes. Doxorubicin, an important component of combination therapy for muscle‐invasive BC, interferes with cell cycle progression and leads to cancer cell death.^49^ Curcumin has a wide range of anticancer activities; it can inhibit the proliferation of BC cells, induce the apoptosis of BC cells, prevent the metastasis of BC cells, promote the antitumor immune response, and enhance the effect of chemotherapeutic drugs when used in combination with chemical drugs.^50^, ^51^, ^52^ However, curcumin has the disadvantage of low bioavailability, and the development of curcumin nanoparticles may significantly improve this problem.^53^ We found that the mRNA levels of CYP1B1 were reduced after curcumin treatment. After doxorubicin treatment, the mRNA levels of MAP1B decreased, and the mRNA levels of ABL2 increased. Therefore, we hypothesized that CYP1B1 and MAP1B are therapeutic targets of these two drugs, while ABL2 is a target of BC cell resistance to doxorubicin; moreover, the underlying mechanism needs further exploration.

Finally, there were several limitations to our study. First, the study used a retrospective design, and there was heterogeneity. Second, our study was still a relatively preliminary bioinformatics study focusing on EC‐related genes in BC. Third, the intrinsic molecular mechanism involved in the interaction between the hub genes and related drugs needed to be explored in depth. In addition, because the clinical information in the TCGA and GEO databases was incomplete and there was a lack of information on some environmental factors and lifestyles, we could not further explore the development mechanism of BC. In summary, we will try our best to construct and integrate cohorts containing more clinical data and epidemiological information in future research to conduct more comprehensive research on the incidence and development of BC. And our findings also need to be verified in future large‐sample multicenter clinical studies and basic molecular biology experiments.

## 5 CONCLUSION

According to the results of the comprehensive analysis, EC‐related genes play a very important role in the diagnosis, prognosis, and treatment of BC, and the related genes are worthy of being targeted for BC treatment. However, incomplete clinical data and a lack of in‐depth mechanistic analysis were limitations of this article. Therefore, future prospective, large‐scale, multicenter studies are needed to confirm our results, and the tumorigenic mechanism of hub genes in BC should be further explored.

## AUTHOR CONTRIBUTIONS

**Yang Fu:** Conceptualization (lead); data curation (lead); resources (lead); software (lead); writing – original draft (lead). **Shanshan Sun:** Conceptualization (equal); formal analysis (equal); resources (equal); software (equal); validation (equal). **Du Shi:** Supervision (lead); validation (lead). **Jianbin Bi:** Writing – review and editing (lead).

## FUNDING INFORMATION

The study was supported by The National Natural Science Foundation of China (Grant No. 82172568).

## CONFLICT OF INTEREST STATEMENT

The authors have no conflicts of interest to declare.

## Supporting information


Figure S1.

Figure S2.

Figure S3.

Figure S4.

Figure S5.

Figure S6.

Figure S7.

Figure S8.

Figure S9.

Table S1.

## Data Availability

The datasets generated for this study can be found in the TCGA database (https://portal.gdc.cancer.gov/), GEO database (GSE13507, GSE32548 and GSE32894; https://www.ncbi.nlm.nih.gov/geo/), TISCH database (http://tisch.comp‐genomics.org/), HPA database (https://www.proteinatlas.org/), PDB database (https://www.rcsb.org/), CTD database (http://ctdbase.org/) and PubChem database (https://pubchem.ncbi.nlm.nih.gov/). All the data from the current study can be acquired from the authors upon reasonable request.
