# Supplementary figures and images for: Construction of endothelial cell signatures for predicting the diagnosis, prognosis and immunotherapy response of bladder cancer via machine learning
Source: J Cell Mol Med. 2024 Mar 1;28(6):e18155. doi: 10.1111/jcmm.18155 (PMC10907833; doi:10.1111/jcmm.18155)

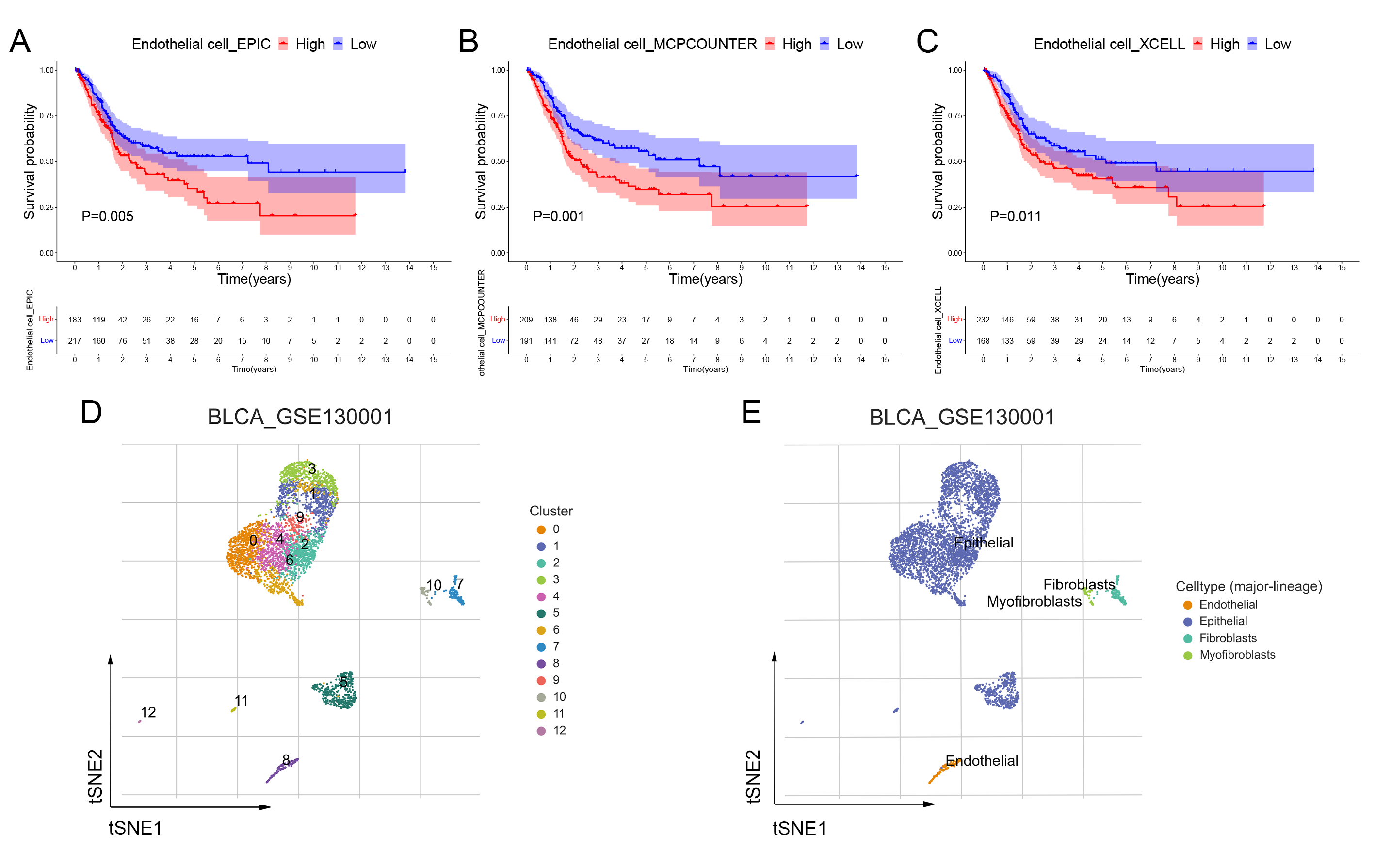

Supplement: Supplementary file 1 — Figure S1. [file JCMM-28-e18155-s002.tif]

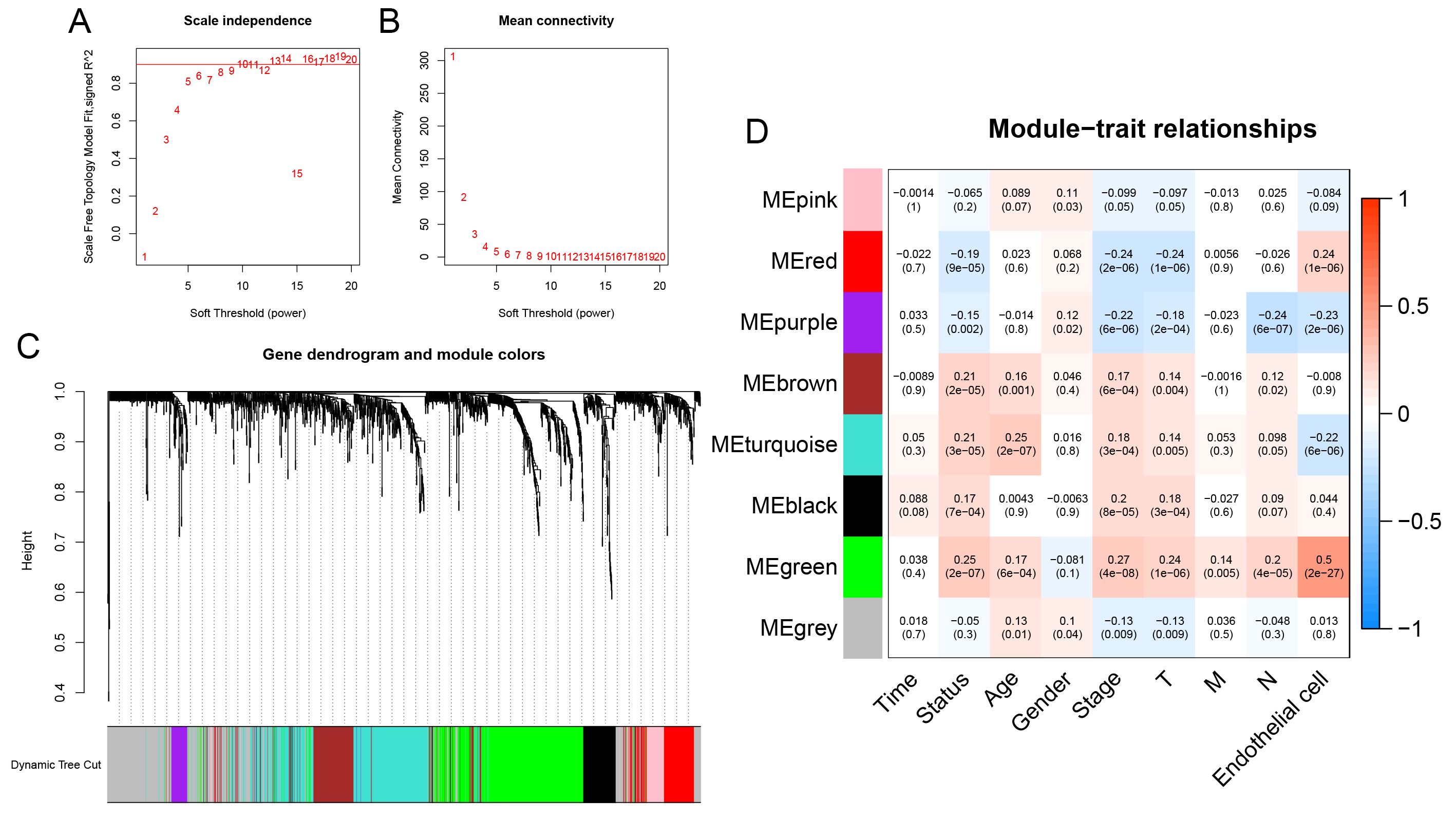

Supplement: Supplementary file 2 — Figure S2. [file JCMM-28-e18155-s003.tif]

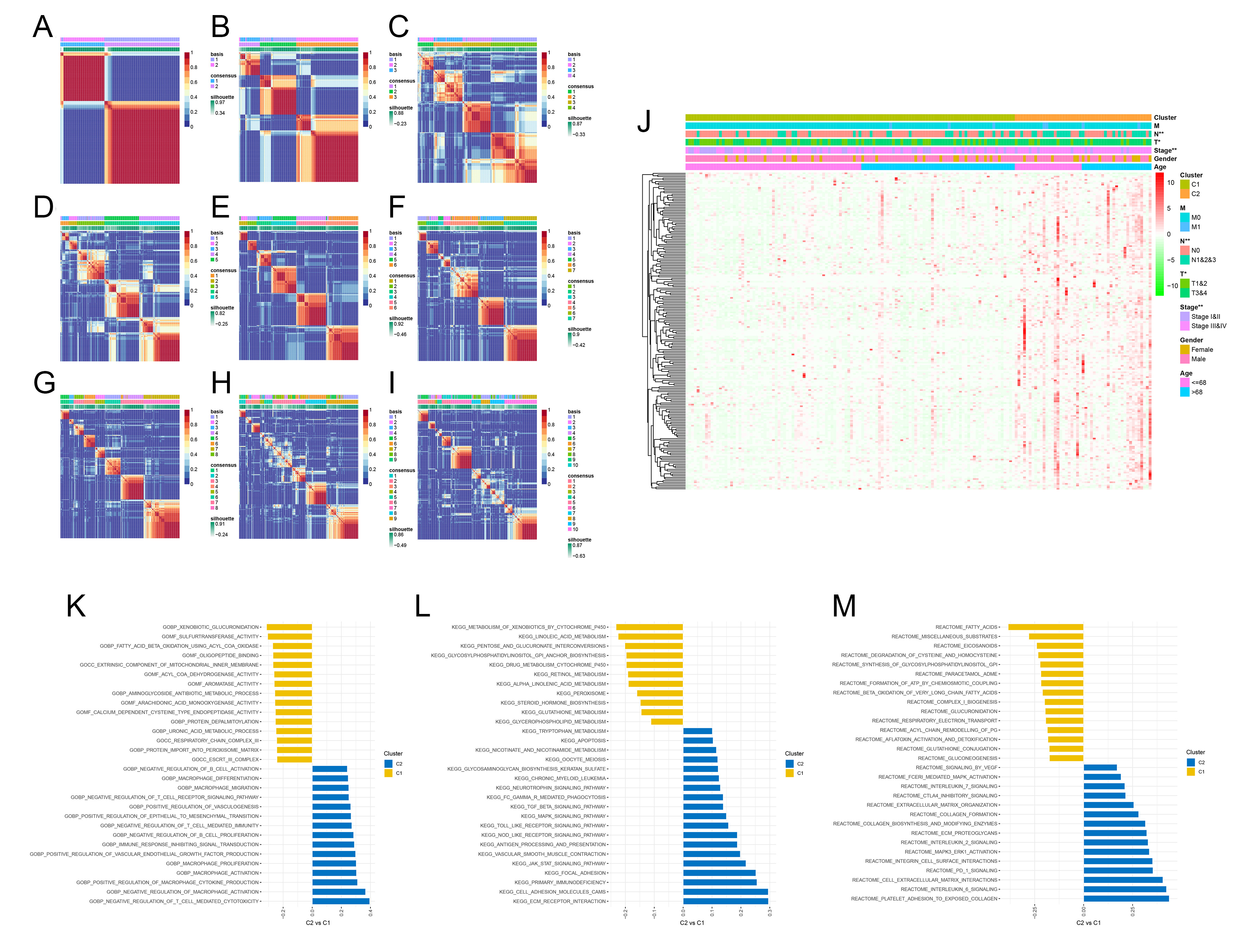

Supplement: Supplementary file 3 — Figure S3. [file JCMM-28-e18155-s007.jpg]

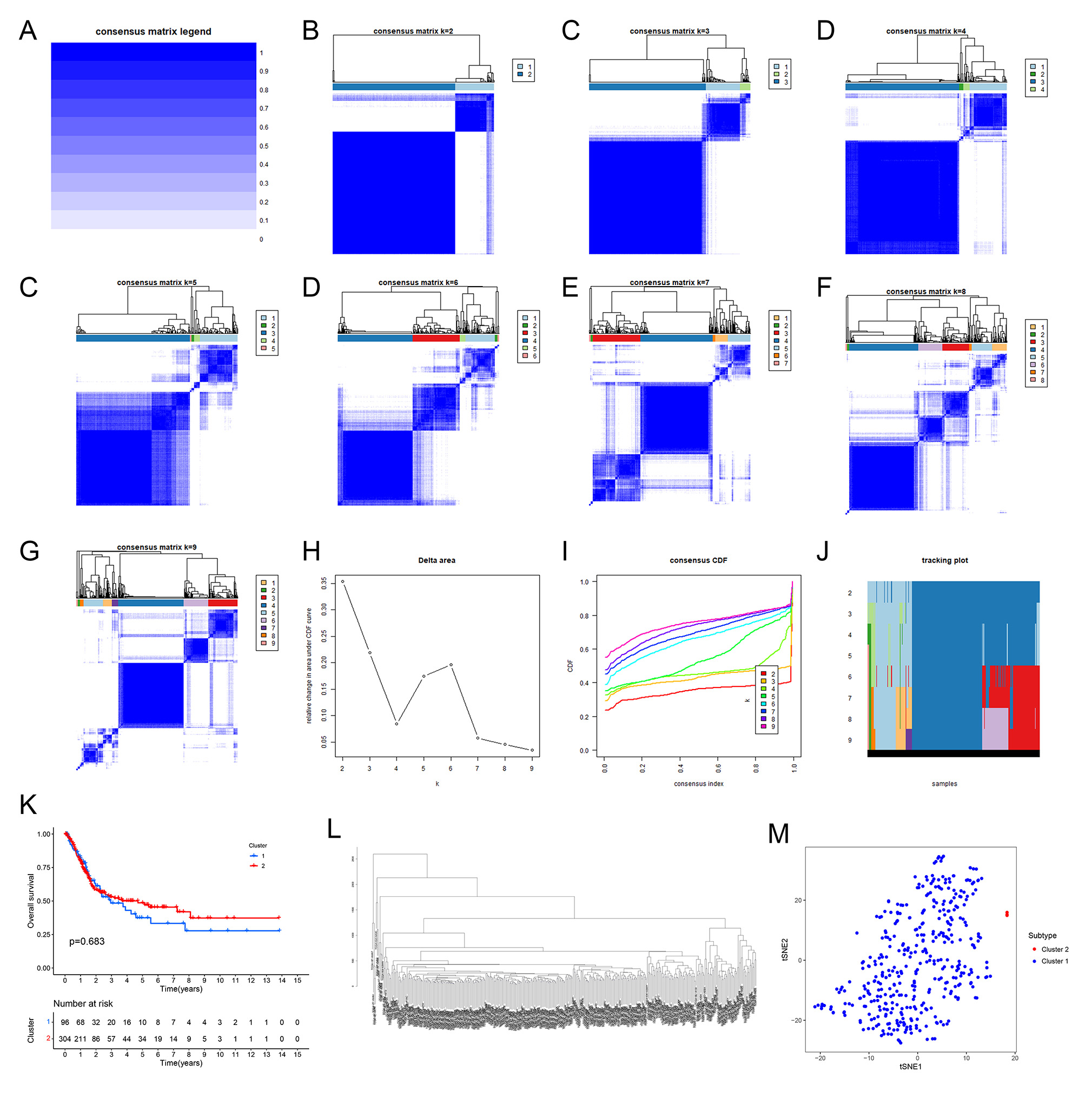

Supplement: Supplementary file 4 — Figure S4. [file JCMM-28-e18155-s004.jpg]

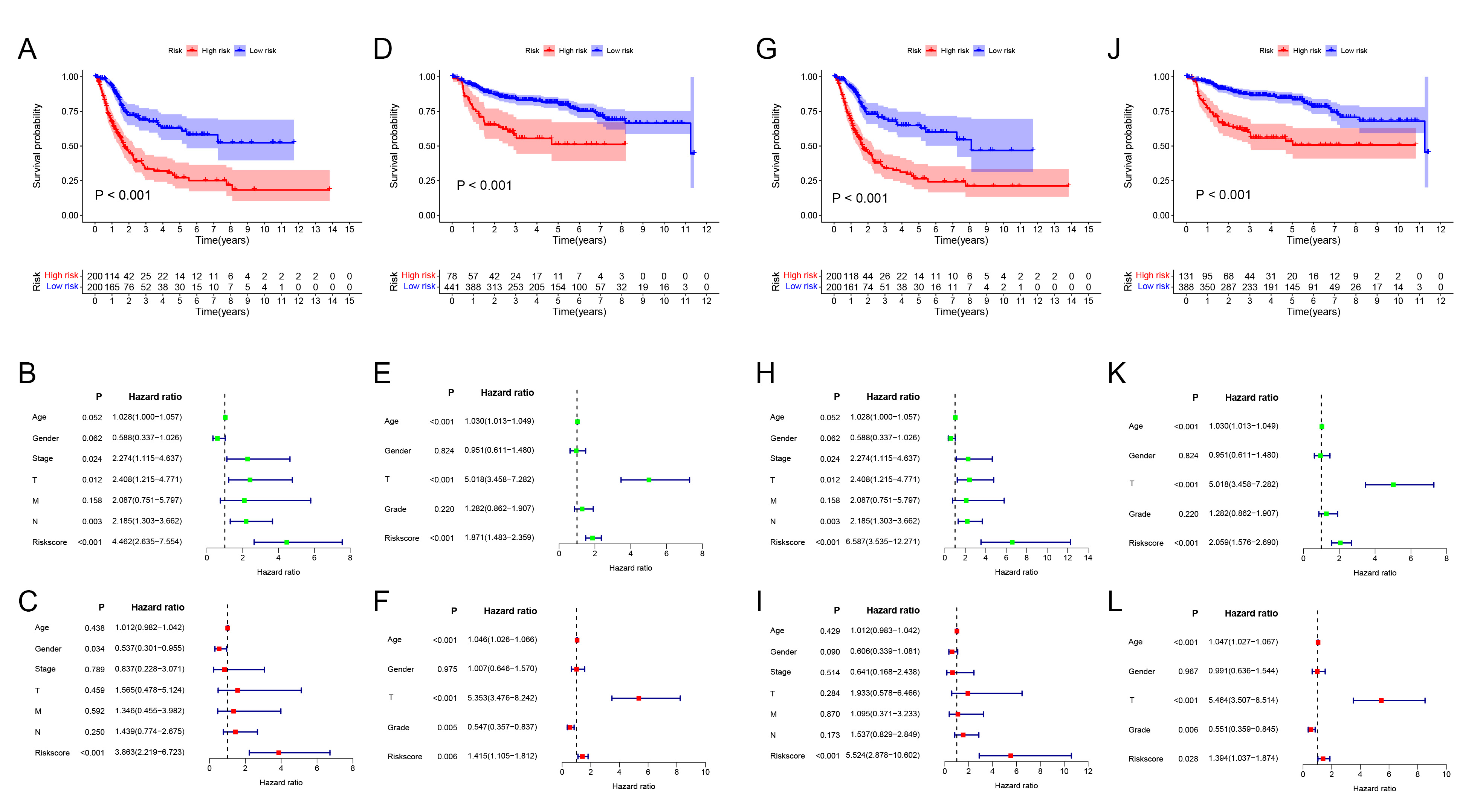

Supplement: Supplementary file 5 — Figure S5. [file JCMM-28-e18155-s001.jpg]

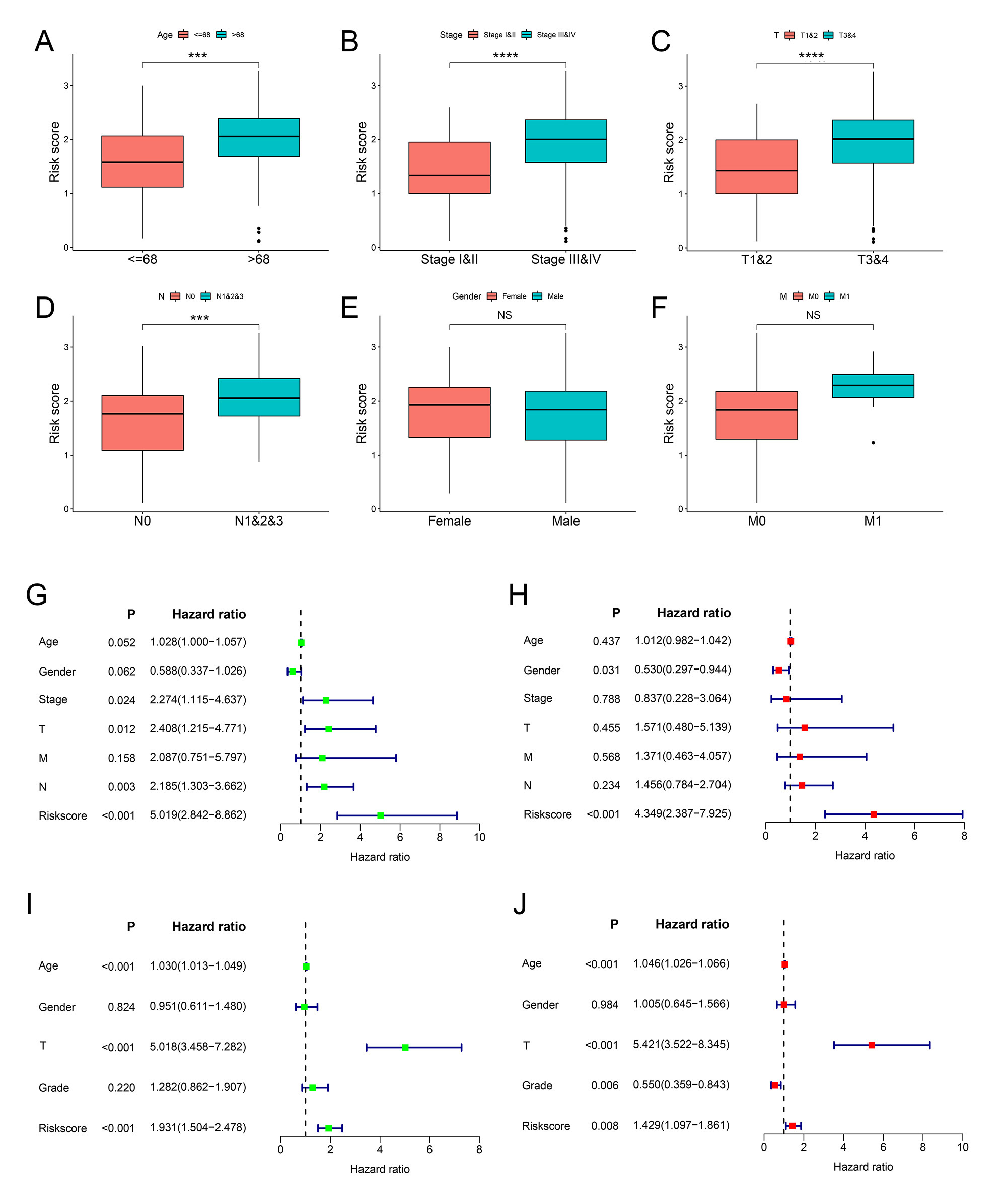

Supplement: Supplementary file 6 — Figure S6. [file JCMM-28-e18155-s010.jpg]

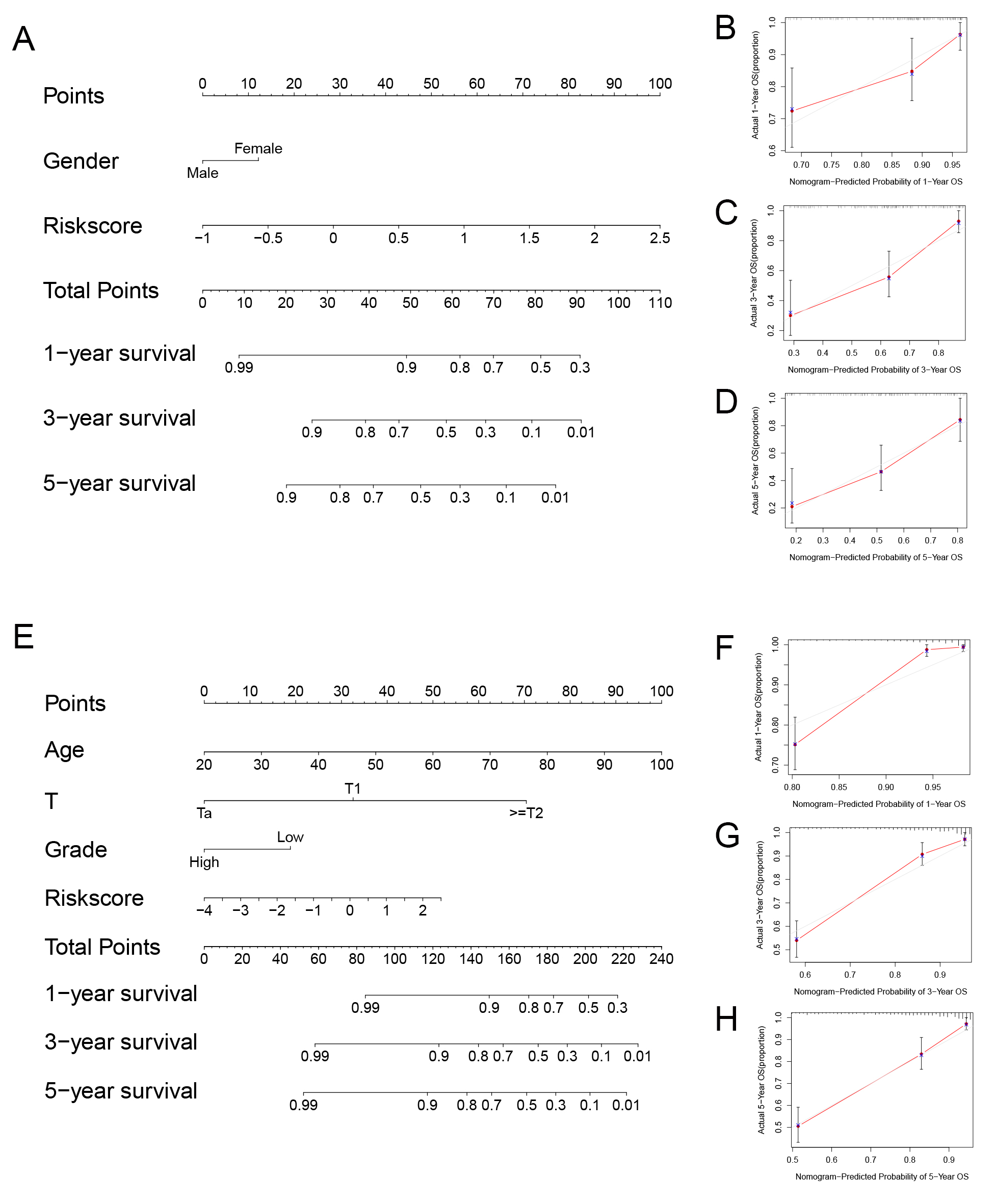

Supplement: Supplementary file 7 — Figure S7. [file JCMM-28-e18155-s008.jpg]

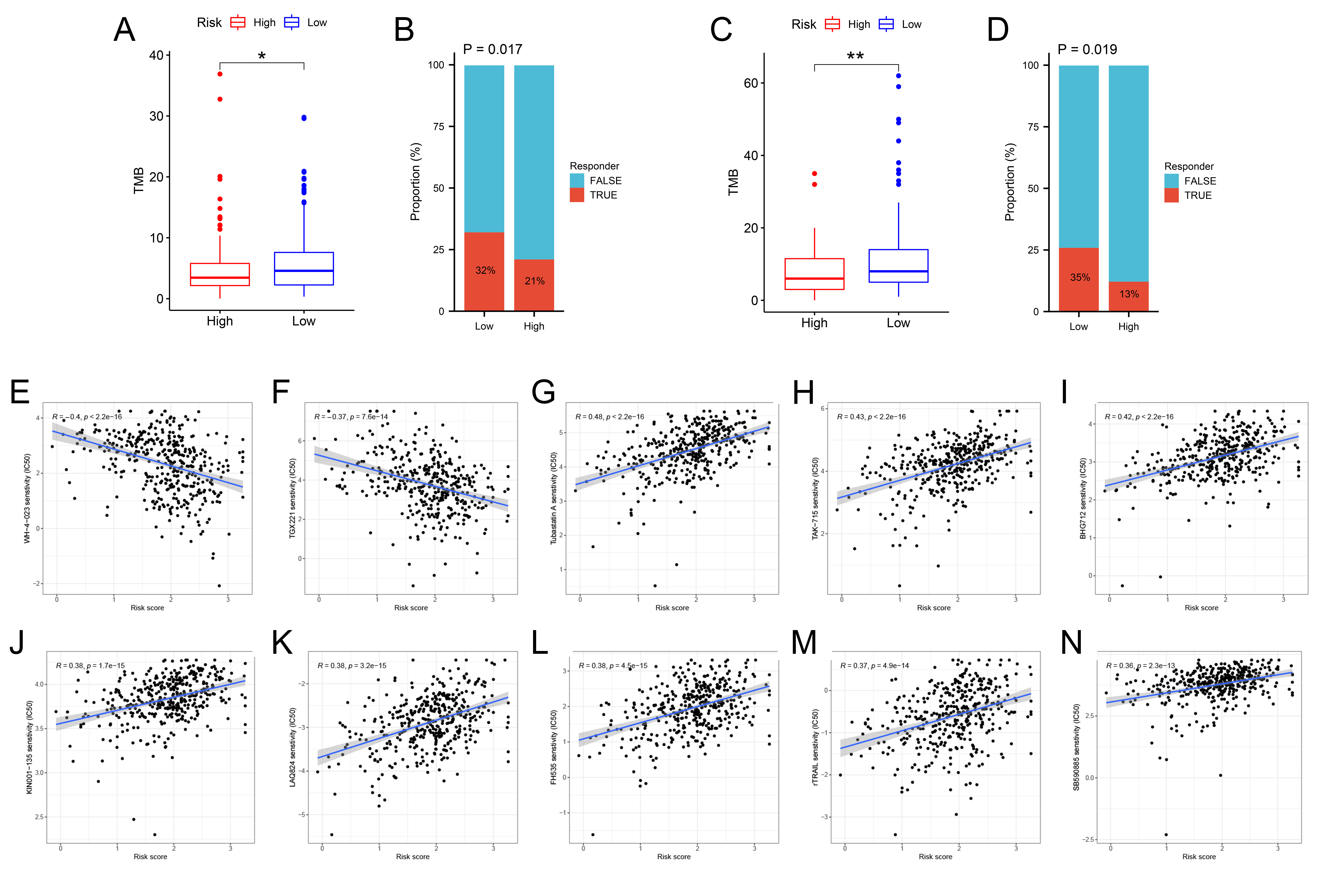

Supplement: Supplementary file 8 — Figure S8. [file JCMM-28-e18155-s005.jpg]

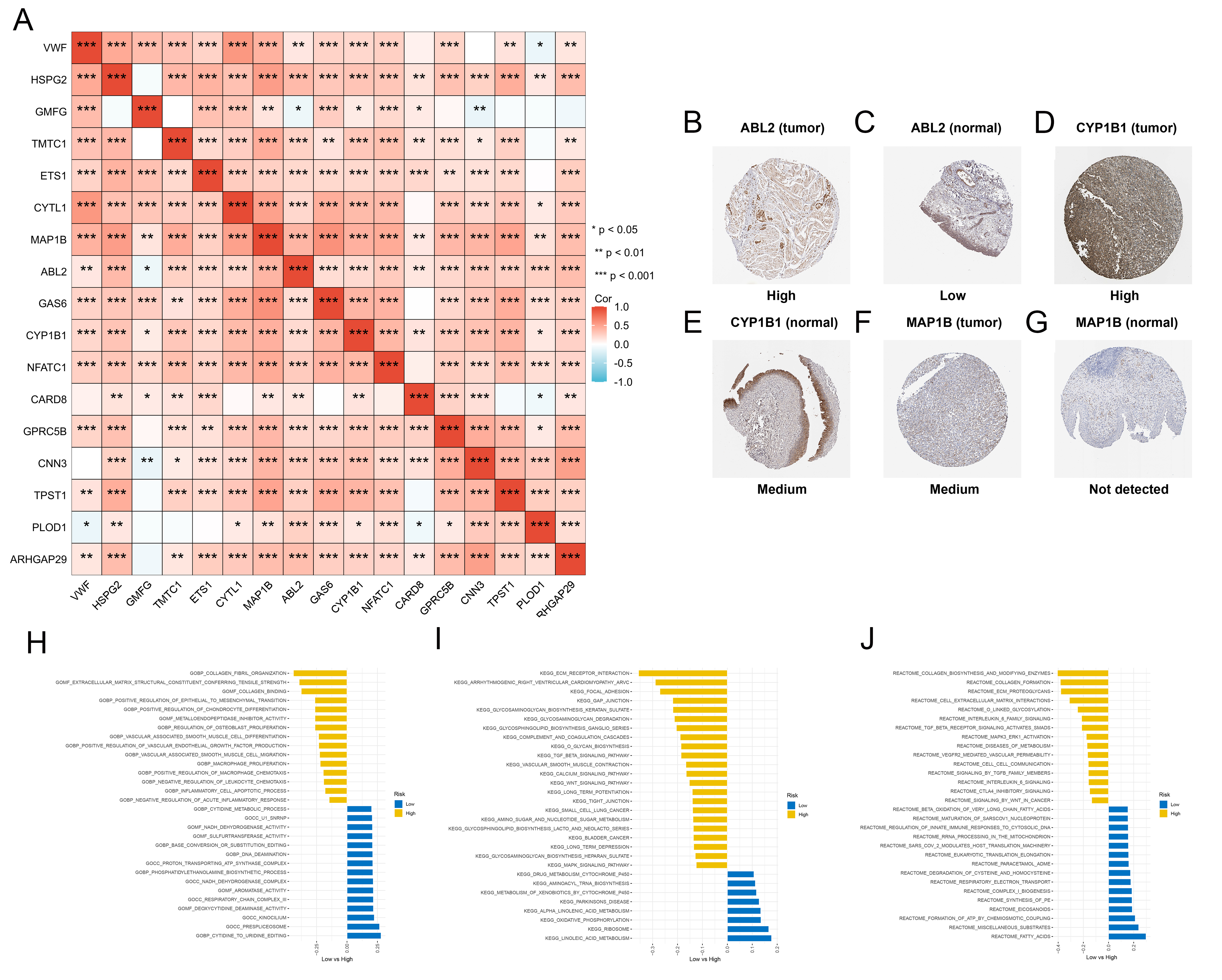

Supplement: Supplementary file 9 — Figure S9. [file JCMM-28-e18155-s009.jpg]
