# Supplementary material for: Construction of endothelial cell signatures for predicting the diagnosis, prognosis and immunotherapy response of bladder cancer via machine learning
Source: J Cell Mol Med. 2024 Mar 1;28(6):e18155. doi: 10.1111/jcmm.18155 (PMC10907833; doi:10.1111/jcmm.18155)
Supplement: Supplementary file 10 — Table S1. [file JCMM-28-e18155-s006.docx]

**Table S1** Genes related to survival were distinguished via univariable Cox regression.

| ID | HR | 95%CI | P value |
| --- | --- | --- | --- |
| ABL2 | 1.882 | 1.203 - 2.943 | 0.006 |
| ADAMTS9 | 1.591 | 1.169 - 2.164 | 0.003 |
| ANXA5 | 1.632 | 1.183 - 2.251 | 0.003 |
| ARHGAP29 | 1.515 | 1.135 - 2.022 | 0.005 |
| BDKRB2 | 1.467 | 1.119 - 1.923 | 0.006 |
| CALD1 | 1.379 | 1.094 - 1.738 | 0.006 |
| CALU | 2.476 | 1.620 - 3.785 | 0.000 |
| CARD8 | 0.402 | 0.236 - 0.684 | 0.001 |
| CCDC80 | 1.437 | 1.167 - 1.769 | 0.001 |
| CD99 | 1.571 | 1.133 - 2.179 | 0.007 |
| CLIC4 | 1.513 | 1.135 - 2.017 | 0.005 |
| CNN3 | 1.983 | 1.397 - 2.814 | 0.000 |
| COL18A1 | 1.533 | 1.147 - 2.049 | 0.004 |
| COL4A1 | 1.454 | 1.095 - 1.931 | 0.010 |
| COL4A2 | 1.575 | 1.177 - 2.106 | 0.002 |
| COL6A2 | 1.305 | 1.072 - 1.589 | 0.008 |
| CTHRC1 | 1.279 | 1.079 - 1.516 | 0.005 |
| CYP1B1 | 1.276 | 1.087 - 1.496 | 0.003 |
| CYTL1 | 1.505 | 1.207 - 1.877 | 0.000 |
| DCHS1 | 1.501 | 1.138 - 1.980 | 0.004 |
| DIXDC1 | 1.641 | 1.225 - 2.199 | 0.001 |
| DPYSL2 | 1.593 | 1.204 - 2.109 | 0.001 |
| ETS1 | 0.619 | 0.451 - 0.850 | 0.003 |
| FAM43A | 1.597 | 1.213 - 2.102 | 0.001 |
| FBN1 | 1.422 | 1.138 - 1.777 | 0.002 |
| FGFR1 | 1.488 | 1.171 - 1.890 | 0.001 |
| FN1 | 1.409 | 1.136 - 1.747 | 0.002 |
| FSTL1 | 1.477 | 1.115 - 1.955 | 0.006 |
| GAS6 | 1.377 | 1.102 - 1.721 | 0.005 |
| GMFG | 0.708 | 0.555 - 0.903 | 0.005 |
| GPRC5B | 1.466 | 1.155 - 1.862 | 0.002 |
| GSN | 1.594 | 1.126 - 2.258 | 0.009 |
| HSPG2 | 1.947 | 1.360 - 2.788 | 0.000 |
| LGALS1 | 1.376 | 1.087 - 1.741 | 0.008 |
| LRRC32 | 1.464 | 1.140 - 1.882 | 0.003 |
| MAP1B | 1.607 | 1.276 - 2.023 | 0.000 |
| MCAM | 1.628 | 1.154 - 2.296 | 0.005 |
| MYADM | 1.369 | 1.084 - 1.729 | 0.008 |
| NES | 1.438 | 1.112 - 1.860 | 0.006 |
| NFATC1 | 1.480 | 1.099 - 1.992 | 0.010 |
| NID1 | 1.559 | 1.174 - 2.071 | 0.002 |
| NRP2 | 1.540 | 1.162 - 2.040 | 0.003 |
| PLOD1 | 1.839 | 1.278 - 2.649 | 0.001 |
| PROS1 | 1.473 | 1.137 - 1.908 | 0.003 |
| PXDN | 1.448 | 1.149 - 1.826 | 0.002 |
| RBP7 | 1.413 | 1.120 - 1.784 | 0.004 |
| SLC2A3 | 1.355 | 1.091 - 1.682 | 0.006 |
| STXBP1 | 1.541 | 1.154 - 2.057 | 0.003 |
| TCF4 | 1.638 | 1.209 - 2.220 | 0.001 |
| TMEM134 | 0.552 | 0.351 - 0.867 | 0.010 |
| TMTC1 | 1.438 | 1.126 - 1.837 | 0.004 |
| TPM1 | 1.498 | 1.152 - 1.949 | 0.003 |
| TPST1 | 2.026 | 1.460 - 2.811 | 0.000 |
| TUBB6 | 1.328 | 1.089 - 1.620 | 0.005 |
| VWF | 1.545 | 1.128 - 2.117 | 0.007 |

HR, hazard ratio; 95%CI, 95% confidence interval.
